# Health-related quality of life in cancer patients treated with immune checkpoint inhibitors: A systematic review on reporting of methods in randomized controlled trials

**DOI:** 10.1371/journal.pone.0227344

**Published:** 2020-01-24

**Authors:** Stéphane Faury, Jérôme Foucaud

**Affiliations:** 1 Laboratory Handicap, Activity, Cognition, Health, EA, University of Bordeaux, Bordeaux, France; 2 INCa, Institut National du Cancer, Boulogne Billancourt, France; Iranian Institute for Health Sciences Research, ISLAMIC REPUBLIC OF IRAN

## Abstract

**Objective:**

Immune checkpoint inhibitors (ICIs) have recently shown tremendous promise in the treatment of diverse cancers. The available data suggests that ICIs are well tolerated in terms of health-related quality of life (HRQOL) compared to other anticancer therapies. However, it appears that instruments currently used to evaluate HRQOL in this context may fail to capture important symptomatology unique to ICIs. This systematic review was designed to assess the adequacy of methods used to report HRQOL in cancer patients treated with ICIs and to identify the quality of life scales used.

**Method:**

A systematic review was performed (systematic registration number: PROSPERO: CRD42019121427). A search of the PubMed, PsycINFO, PsycARTICLES, Psychology and Behavioral Sciences collection, and SocINDEX databases was carried out for publications in English and in French. Relevant databases were searched from the earliest records through to March 2019. Publications were selected if they reported on HRQOL in patients with cancer treated by ICIs. Risk of bias was scored using the Cochrane Collaboration bias assessment tool.

**Results:**

Our search identified 144 publications between 2012 and 2019, of which 15 RCTs met the inclusion criteria. The results suggest that even though the overall reporting of HRQOL was deemed to be of good quality, the data available was marred by methodological aspects such as the lack of HRQOL research hypotheses and the lack of questionnaires validated for cancer patients treated with immunotherapy.

**Conclusion:**

This study provides a comprehensive analysis of the current state of the art and identifies gaps in knowledge on HRQOL analysis with respect to ICIs. It also suggests avenues for further research.

## Introduction

Cancer immunotherapy has revolutionised the treatment of cancer and represents a new option for clinicians [1–3]. Immune Checkpoint Inhibitors (ICIs) have garnered attention as one of the most promising types of immunotherapy [4,5]. The U.S. Food and Drug Administration (FDA) approved seven ICIs for clinical use: Ipilimumab, Pembrolizumab, Nivolumab, Atezolizumab, Durvalumab, Avelumab and Cemiplimab [6].

Checkpoint inhibition is associated with a unique spectrum of side effects known as immune-related adverse events (irAEs), defined as toxicities caused by non-specific activation of the immune system [7, 8, 9]. Some irAEs can be serious and even life-threatening, and thus substantially affect patients’ daily lives [10]. Abdel-Rahman et al. (2018) performed a systematic review to explore time to HRQOL deterioration among cancer patients receiving PD-(L)1 inhibitors compared to those receiving traditional cytotoxic therapy. The results showed that PD-(L)1 inhibitors were associated with a consistent prolongation of the time to symptomatic deterioration [11]. Recently, Hall et al. (2019), in their systematic review, suggested that ICIs are well tolerated in terms of HRQOL compared to other anticancer therapies. However, for these authors, currently used instruments may fail to capture important symptomatology unique to ICIs [12]. For example, in the CheckMate141, trial of Nivolumab *versus* therapy of investigator’s choice in recurrent or metastatic head and neck, the QLQ-H&N35 was applied to evaluate HRQOL. But skin toxicities (e.g. o, L er). QOL e prominent role as important endpoints in cancer RCTs.atuc review to i …ndart therapy arm (t not be covered.contsuchrash and pruritus), the most frequent irAEs with Nivolumab [8,13], are not assessed with this HRQOL measure [13]. Results of Hall et al. (2019) should therefore be interpreted with caution.

To date, HRQOL refers to a multidimensional concept including the domains of physical, emotional and social functioning [14]. Assessing HRQOL in cancer patients is necessary to evaluate the full impact of the cancer experience [15] and to improve understanding of how different therapeutic interventions influence patients’ outcomes [16]. HRQOL is increasingly recognized as an essential end point in cancer clinical trials [17]. HRQOL outcomes from RCTs increasingly inform cost-effectiveness analyses used by policymakers to decide on the allocation of resources [18,19]. Therefore, it is imperative that findings from RCTs are robust.

HRQOL evaluations are made using standardised and validated self-assessment methods [20]. The most important methodological issue to consider in evaluating HRQOL endpoints in an oncology clinical trial is the selection of appropriate outcome measurements [21]. There is also evidence that the potentially invaluable insights that HRQOL data provide on the treatment and care of patients may not be adequately reported [19,22–24].

To our knowledge, the reporting of quality-of-life methods in trials of cancer patients treated with ICIs has not been systematically assessed. The following systematic review aims to evaluate the quality of HRQOL methodological assessment used to evaluate the effects of ICIs on HRQOL in randomised controlled trials (RCTs) to determine how improvements can be made, and to explore the value added by these measures to clinical decision-making in a trial setting. We focused on RCTs they represent the gold standard for evaluating new medical treatments, developing decision-making policies, and planning new treatment approaches [25,26].

## Method

In accordance with French regulations, this study was exempted from IRB approval. The methods discussed in this review were previously published in the PROSPERO database [27], under number CRD42019121427. (http://www.crd.york.ac.uk/PROSPERO/display_record.php?ID=CRD42019121427).

### Search strategy

We used standard procedures: Preferred Reporting Items for Systematic reviews and Meta-Analyses (PRISMA) guidelines [28,29], in accordance with the principles outlined in the Cochrane Handbook for Systematic Reviews of Interventions [30]. A systematic literature search was performed from 1^st^ March 2019; we searched for all RCTs published up to 28^th^ February 2019, irrespective of their start or completion date. We searched PubMed, PsycINFO, PsycARTICLES, Psychology and Behavioral Sciences collection, SocINDEX, limiting the search to RCTs of adults (≥ 18 years at diagnosis) published in English or French, and confined to original human studies published in peer-reviewed journals. Companion papers that focused only on HRQOL were reviewed with the original publication.

We identified relevant references using the following terms: “quality of life”, “cancer”, “neoplasms” and individual drug names. The reference lists of key papers were checked to find relevant references for inclusion. Note that, when we started our research (March 2019), the Cemiplimab had been recently approved by the FDA (September 2018). It was not specifically examined in our search strategy due to the lack of data on HRQOL available in March 2019 for this ICIs (clinicaltrials.gov).

The full search algorithm, based on Hall et al. [12], used to identify potential studies in PubMed is included in S1 Search algorithm and was adapted for the other databases.

### Selection criteria

The patient population assessed included patients with any cancer randomised to treatment with an approved ICI. Predefined exclusion criteria were: RCTs that assessed treatment that did not include ICIs, RCTs on patients with other illnesses, phase I studies, and RCTs with fewer than ten patients per group. As a single domain (e.g. fatigue) is not considered as HRQOL, trials assessing only one aspect of HRQOL were excluded. Companion papers focusing on a subgroup of the total RCT sample were excluded. Unpublished reports, conference abstracts, and dissertations were excluded due to the lack of peer-review oversight. These are often subsequently published in peer-reviewed journals, risking duplication.

### Data extraction

An extensive search of electronic databases was conducted by one person (SF). Literature search results were uploaded to Zotero (www.zotero.org) which facilitates bibliographic source management. Following the removal of duplicates, two reviewers (SF and JF) independently screened titles and abstracts of all references identified by the search according to eligibility criteria. Full articles were obtained for all titles that either appeared to meet the inclusion criteria or where uncertainty as to the eligibility criteria existed. A final selection pertaining to the suitability of the full-text papers to be analysed was verified by two reviewers (SF and JF). A third reviewer (CC) was available as a mediator in the event of disagreement.

### Data analysis

The criteria used to evaluate quality of reporting on HRQOL were based on those proposed by Efficace et al. [25]. On the basis of good practice in reporting HRQOL [24, 31–34], Efficace et al. [25] extracted 11 basic and essential issues that a given trial should report to reach methodologically sound outcomes. The checklist items were devised to have a dichotomous answer: these can be scored as ‘yes’ (giving a score of 1) or ‘no’ (giving a score of 0), with higher scores indicating the robustness of the outcomes. The items included in the checklist are self-explanatory and a brief description is also provided in S1 Table.

### Determining study quality

Risk of bias was assessed by two reviewers using the Cochrane Collaboration bias assessment tool [35]. Responses in each domain (random sequence generation, allocation concealment, blinding of participants and personnel, blinding of outcome assessment, incomplete outcome data and selective outcome reporting) were assessed as having a ‘low’, ‘unclear’ or ‘high’ risk of bias.

## Results

### Key characteristics of identified trials

**Fig 1:**
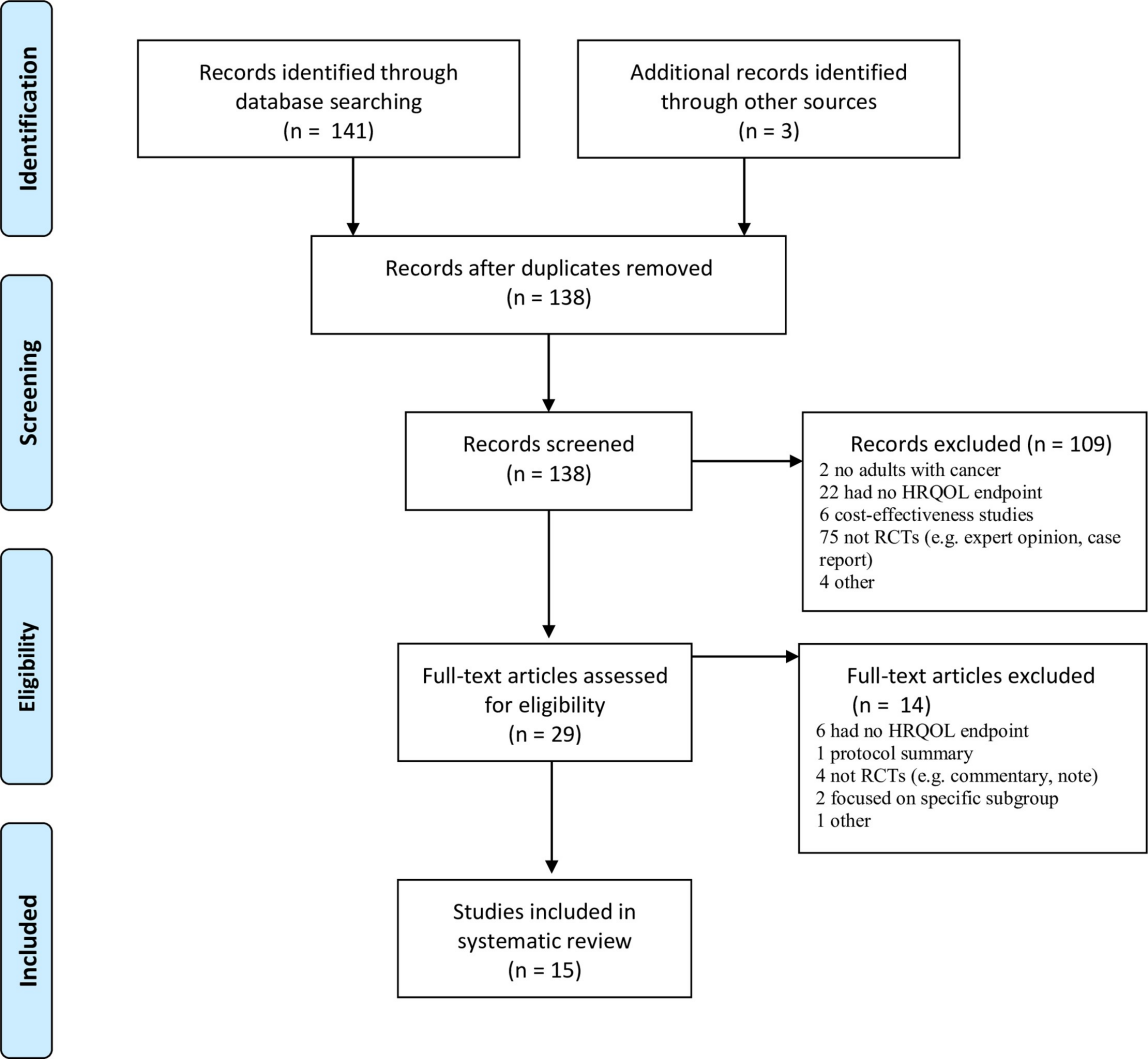
Selection strategy–study inclusion and exclusion flowchart. HRQOL = health-related quality of life. RCTs = randomized controlled trials. ICIs = immune checkpoint inhibitors. * = some RCTs included more than one ICI.

The literature search yielded 144 records. After removal of duplicates, the titles and abstracts of 138 records were screened; 109 records were identified as clearly non-relevant and consequently excluded: full-text articles were thus obtained for 29 papers. Fourteen of these publications were excluded as not meeting the selection criteria (e.g. lack of HRQOL endpoint, protocol summary, RCTs, specific subgroup of RCTs). This gave a total of 15 publications to be included in the systematic review (Fig 1). Publications covered a seven–year period (2012–2019). The key demographic characteristic results from the 15 RCTs included in this systematic review are summarised in Table 1.

**Table 1:** Key characteristics of 15 RCTs between 2012 and 2019.

	Number of RCTs	
	*n*	*%*
**Total number of reviewed RCTs**	15	100
Number with HRQOL as primary endpoint	0	0
Number with HRQOL as secondary endpoint	9	60
Number with HRQOL as exploratory endpoint	5	33
Number with additional HRQOL publication	13	87
Number discussing HRQOL in the main publication	7	47
Multi-country locations	15	100
Industry funded	15	100
Type of ICI*		
Atezolizumab	1	7
Avelumab	0	0
Durvalumab	0	0
Ipilimumab	5	33
Ipilimumab + Nivolumab	2	13
Nivolumab	7	47
Pembrolizumab	4	27
Non-checkpoint inhibitor controls	12	80
Chemotherapy	8	53
Placebo	1	7
Everolimus	1	7
GP100	1	7
Sunitinib	1	7
Type of cancer		
Melanoma	8	53
Non-Small Cell Lung Cancer	3	20
Squamous Cell Carcinoma of the Head and Neck	1	7
Advanced renal cell carcinoma	2	13
Urothelial cancer	1	7
Sample size		
272 to 500	5	33
501 to 800	5	33
801 to 951	5	33
Peer-reviewed clinical journals		
N Engl J Med (IF = 72.406)	1	7
Lancet Oncol (IF = 33.900)	5	33
J Clin Oncol (IF = 26.303)	2	13
Ann Oncol (IF = 11.855)	1	7
J Thorac Oncol (IF = 6.595)	1	7
Eur J Cancer (IF = 6.029)	3	20
Clin Lung Cancer (IF = 3.66)	1	7
Health Qual Life Outcomes (IF = 2.143)	1	7

RCTs = randomised controlled trials. HRQOL = health-related quality of life. ICI = immune checkpoint inhibitors.

* = Some RCTs used more than one ICI.

Nine of the 15 RCTs were published in high-impact journals (> 10). For 13 of the 15 identified RCTs, the HRQOL data were published in a companion paper. Results of HRQOL data are not presented in the main papers of these 13 RCTs [36, 37, 38, 39, 40, 41, 42, 43, 44, 45, 46, 47, 48]. Eight of the thirteen HRQOL companion papers were published in the year after publication of the main clinical results, four within two years, and one within three years. All trials were industry-sponsored and/or affiliated with the pharmaceutical industry through one or more investigators, as noted in the financial sponsorship statements of the articles. All trials were multicentre trials and performed in more than one country. In total, 9332 patients were enrolled in the 15 RCT trials. Trial size ranged from 272 to 951 patients. The ICIs studied in these RCTs were Ipilimumab (33%), Pembrolizumab (27%), Atezolizumab (7%) and Nivolumab (47%). No relevant RCTs with HRQOL assessment focused on Durvalumab or Avelumab. The non-checkpoint inhibitor controls used in some of the studies included conventional treatment, chemotherapy, sunitinib, placebo, or everolimus, and gp100. Eight RCTs assessed ICI treatments for melanoma, three for lung cancer, one for head and neck squamous cell carcinoma, one for urothelial cancer, and two for advanced renal cell carcinoma. All trials had overall survival or progression-free survival as the primary endpoint; all reported significant differences in one of these endpoints between treatment arms. HRQOL was a secondary endpoint in nine trials [49,50,51,52,53,54,55,56,57], an exploratory endpoint in five RCTs [58,59,60,61,62] and for one trial it was unclear if the HRQOL endpoints were secondary or exploratory [63]. Seven trials discussed HRQOL in their main publication [50,59,60,52,61,56,57].

### Quality of HRQOL measurements

The conceptual measurement and methodology for evaluating HRQOL outcomes from the 15 RCTs included in this systematic review are summarised in Table 2 and S2 Table. HRQOL was measured with common cancer HRQOL assessment tools in all of the studies. The European Organisation for Research and Treatment of Cancer (EORTC) Quality of Life Questionnaire (EORTC QLQ-C30, used in twelve of the 15 RCTs), and the European Quality of Life 5 Dimension (EQ-5D, used in eleven of the 15 trials) were the most frequently used. In two of the twelve studies, the EORTC QLQ-C30 was supplemented with the EORTC Quality of Life 13-item Lung Cancer-specific Questionnaire (EORTC QLQ-LC13), and, in another, with the EORTC module for Head & Neck cancer (EORTC QLQ-H&N35). Additional HRQOL instruments used included the Functional Assessment of Cancer Therapy (FKSI-DRS or FKSI-19, used in two trials), the Functional Assessment of Cancer Therapy-General (FACT-G, used in one trial), and the Lung Cancer Symptom Scale (LCSS, used in one trial). Twelve trials used more than one HRQOL questionnaire.

**Table 2:** Level of reporting according to the minimun standard checklist for evaluating HRQOL outcomes in cancer clinical trials.

HRQOL Issue	Reports	
	No.*	%
Conceptual		
A priori hypothesis stated	5/10	50
Rationale for instrument reported	13/15	87
Measurement		
Psychometric properties reported	14/15	93
Cultural validity verified	4/15	27
Adequacy of domains covered	0/15	0
Methodology		
Instrument administration reported	8/15	53
Baseline compliance reported	14/15	93
Timing of assessment documented	15/15	100
Missing data documented	15/15	100
Interpretation		
Clinical significance addressed	13/15	87
Presentation of results in general	13/15	87

HRQOL: Health-Related Quality Of Life.

* Number of articles reporting item/number of articles to which item is applicable.

Five of the 15 RCTs did not specify the HRQOL research hypothesis due to the HRQOL endpoint being an exploratory objective [58,59,60,61,62]. For Efficace et al. [25], to satisfy the “*a priori hypothesis stated*” criterion, the study needed to predefine HRQOL end point and/or state expected changes because of the specific treatment. Only five trials of the 15 RCTs satisfied this criterion but none of them included an *a priori* hypothesis for the expected HRQOL outcomes [49,50,51,54,55].

Two of the 15 studies failed to report their reasoning for using a specific HRQOL measure and none gave a reason for choosing a particular HRQOL instrument. For 14 trials, the validity and reliability (“psychometric properties reported” criterion) of the instruments used was reported by referencing the appropriate validation studies; one trial provided no reference to validity or reliability [62]. All but one of the questionnaires (EQ-5D) were validated for the specific cancer population (“Cultural validity verified” criterion). The EQ-5D is a non-cancer-specific measure of generic health for clinical and economic appraisal. None of the questionnaires used in these 15 trials (EORTC questionnaires, EQ-5D, LCSS, FKSI, FACT-G, etc.) were validated among cancer patients treated with ICIs (“adequacy of domains covered” criterion). Checkpoint inhibition is associated with a unique spectrum of side effects that are not assessed with the HRQOL measure used. The HRQOL domains covered by the questionnaires were inadequate for several of the trials identified. Details describing how the HRQOL assessment was done were often not reported. Some details were noted for only eight of the trials (“instrument administration reported” criterion). All the RCTs reported the timing of HRQOL assessment and documented missing data.

We observed that presentation of the HRQOL results was adequate for all but two of the trials. For these two trial presentations of HRQOL, the results were defined as limited (main publication of RCTs), with incomplete HRQOL details and a discussion of the HRQOL outcomes in terms of clinical significance not reported. HRQOL data was judged as high quality if at least eight of the 11 criteria were satisfied; furthermore, three of the eight or more criteria satisfied needed to cover the three high-priority concerns identified by the experts (“baseline compliance reported,” “psychometric properties reported,” and “missing data documented”). If one or two of the 11 items on the checklist were assessed as not applicable, the cutoff was set at seven or six criteria respectively (although the three mandatory criteria still had to be met). Only for ten trials of the 15 RCTs HRQOL data was judged as high quality [49,58,50,59,51,60,52,54,55,63]. The others trials were considered to have some possible reported methodological limitations because of studies addressed (1) at least eight (or seven if one of the 11 items on the checklist were assessed as not applicable) issues but did not take into account the mandatory items [52, 61, 62]; (2) fewer than eight (or seven) issues [56,57].

### Risk of bias

**Fig 2:**
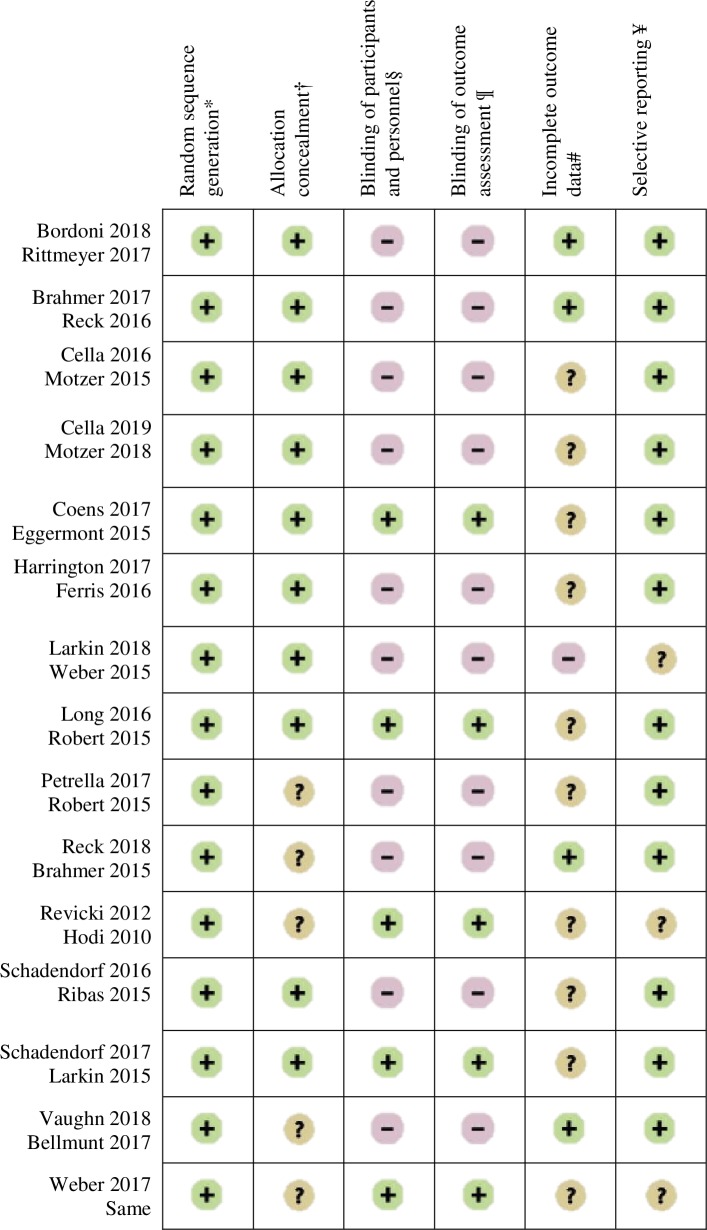
Risk of bias. + = low risk of bias. − = high risk of bias. ? = unclear risk of bias. * = bias due to inadequate generation of a randomised sequence. † = bias due to inadequate concealment of allocations before assignment. § = bias due to knowledge of the allocated interventions by participants and personnel during study. ¶ = detection bias due to knowledge of the allocated interventions by outcome assessment. # = due to amount, nature or handling of incomplete outcome data. ¥ = bias due to selective outcome reporting.

Fig 2 summarises the risk of bias for all of the RCTs evaluated. All the RCTs used block randomisation and/or stratified randomisation to generate the random sequence and were assessed as having a ‘low’ risk of bias for random sequence generations. For ten RCTs, participants were randomly assigned by an interactive voice response system. These trials were assessed as having a ‘low’ risk of bias for allocation concealment. The five other studies were assessed as having an ‘unclear’ risk of bias for this item. Ten RCTs were open-label (trial in which both the researchers/investigators and participants were not blinded to treatment allocation). This procedure could affect patients’ perceptions of HRQOL. For these ten open-label RCTs, the blinding of outcome assessment and blinding of participants and personnel were assessed as having a ‘high’ risk of bias. The five other RCTs were double-blind trials and were assessed as having a ‘low’ risk of bias for these two items. Four RCTs were assessed as ‘low’ risk of bias and ten as having an ‘unclear’ risk of bias for attrition criteria. For twelve RCTs, pre-specified HRQOL outcomes were reported and were assessed as having a ‘low’ risk of bias for reporting bias. The key reason for the ‘unclear’ risk of bias assessment was the lack of method details.

## Discussion

Introduced in the past ten years, immunotherapy is starting to revolutionize the treatment of cancer. ICIs are increasingly used to treat a variety of cancers, but little is known about HRQOL. Hall et al. (2019) [10] performed a systematic review to examine HRQOL among cancer patients receiving ICIs as compared to other anticancer therapies: they did not analyse investigate the HRQOL assessment methods used in RCTs on cancer patients treated with the ICIs and it is the purpose of our work.

Fifteen RCTs meeting the inclusion criteria were identified in the literature. None of the identified RCTs studied Durvalumab or Avelumab HRQOL, probably as a result of delay in publishing data. Our analysis showed that all included RCTs were international, multicenter and supported by commercial sponsors. The primary endpoint of all of the RCTs was efficacy and safety, using progression-free survival or overall survival. HRQOL was identified as a secondary, or even exploratory, endpoint. Overall, a recent systematic review [64] about quality of patient-reported outcome (PRO) reporting across 557 cancer RCTs showed that PRO were secondary endpoints in 421 RCTs (76%). About HRQOL endpoint, RCTs on cancer patients treated with ICIs are in line with RCTs across cancer patients. For most of the RCTs, the HRQOL data were published in a companion paper in a peer-reviewed journal. Most of the companion papers were published within two years after publication of the main clinical results. The number of additional HRQOL papers is significant and indicative of interest not only for the efficacy of new drugs, but also for the patients’ experience.

Previous studies [11,12] showed that patients receiving ICIs experience similar-to-improve HRQOL compared to patients receiving other treatments for advanced cancer (such as chemotherapy, placebo, everolimus or gp100). Based on our findings, these results must be interpreted with caution in view of a number of potential weaknesses associated with the RCTs included.

First, as there are no international guidelines concerning the statistical analysis of HRQOL data in cancer RCTs [65], a clear ‘*a priori* hypothesis’ is needed to develop an analysis plan [20]. In Efficace et al. (2015) systematic review [64], about PRO reporting across 557 cancer RCTs, the three items most frequently omitted from reports were (1) the statement of a PRO hypothesis, (2) the description of statistical approaches for dealing with missing PRO data, and (3) the documentation of methods for PRO data collection, which were reported in only 17%, 20%, and 24% of studies, respectively. In line with the results of Efficace et al. (2015), the ‘stated *a priori* hypothesis’ is the most important HRQOL assessment method that needs to be implemented in RCTs about patients treated with ICIs. Indeed, none of the trials included an *a priori* hypothesis for expected HRQOL outcomes. As already mentioned [10,20,22,25], defining and reporting a hypothesis is an essential requirement for an appropriate study design. The choice of HRQOL questionnaire and statistical analysis in RCTs is dependent on the objective. A clear objective and specific hypotheses improve the analysis and credibility of the results [10]. The lack of hypotheses in many RCTs might partly be due to the exploratory nature of the analysis of HRQOL. Failure to pre-specify hypotheses might generate spurious HRQOL results, potentially emphasising findings that are statistically, but not clinically, relevant [25]. Consequently, we should be careful to avoid interpreting the HRQOL results of previous studies [11,12] as if based on a confirmatory analysis.

Second, as the choice of instrument affects reporting, analysis and interpretation of the trial findings deserve adequate justification [20]. In theory, both generic and disease-specific questionnaires may be used for a given condition. In practice, it is very important to choose the most suitable questionnaire to explore the domains relevant to the treatment(s) [66]. In our systematic review, while the characteristics of the HRQOL instrument used were presented for all trials but one, none of them justified their choice of a particular HRQOL instrument. As the questionnaires used in the RCTs included are considered by researchers to be the standard for use in cancer RCTs, they are often reported without a justification for their particular selection. The lack of a stated *a priori* hypothesis plays a part in the choice of the instrument as justification for the use of a specific method should depend mainly on the hypothesis being examined [20]. All but one of the questionnaires (EQ-5D) were validated for the specific cancer population. Nevertheless, none of these, or their clinically meaningful cut-off, were validated for cancer patients treated with immunotherapy. This is of the utmost importance as ICIs have a different toxicity profile to chemotherapy [20]. For example, skin toxicities (e.g. o, L er). QOL e prominent role as important endpoints in cancer RCTs.atuc review to i …ndart therapy arm (t not be covered.contsuchrash and pruritus) are the most frequent irAEs with anti-PD1 [8,13], but skin problems are not assessed with QLQ-H&N35. Consequently, HRQOL might be affected by ICIs in domains that are not covered by standard instruments, whereas side effects of chemotherapy (for control groups of RCTs) are covered by these standard instruments. Moreover, for the QLQ-C30, for example, although mean score changes of 10 points are widely viewed as clinically significant [67], minimally important differences as low as 4 points have been reported in other cancer trials [68,69]. When interpreting the trial results, it should be kept in mind that important HRQOL issues might not be covered [70].

Furthermore, quality of life may be defined as an individual’s self-perceived satisfaction at any stage of life [71]. Since the disease and cure are associated with symptoms and side effects, the patient learns to adapt to them [72]. This may change the patient’s internal standard of assessment. Such changes indicate the phenomenon of ‘response shift’ [73,74]. Thus, measurement of HRQOL changes may be biased [75–77]. None of the RCTs measured this bias, although different methods can be used to measure the phenomenon (e.g., Then-test method, Ideal Scale approach, Schedule for the Evaluation of Individual Quality of life).

Moreover, human behaviour is influenced by what we know or believe. The common sense model of self-regulation of health and illness (CMS) proposed by Leventhal (1980) [78] postulates that individual perceptions of health threats, and the ensuing emotional response, guide peoples' coping responses. CSM classifies illness representations into distinct dimensions: identity, cause, timeline, consequences, personal control, treatment control and illness coherence. In their systematic review and meta-analysis, Richardson et al. demonstrated that lower identity scores (referring to the description of the health threat and its symptoms) were associated with better quality of life [79]. In research, there is a risk of expectations influencing findings, most obviously when there is some subjectivity in the assessment, as in HRQOL, leading to biased results. To eliminate such bias, patients and assessors need to be blinded to treatment allocation [80]. In our systematic review, ten RCTs were open-label, and patients' perception of their quality of life could have been influenced by their knowledge of the treatment received. Most RCTs used centralised treatment allocation (interactive voice response system) and reported that the method adopted to stratify patients can help prevent potential treatment imbalances. However, instrument administration was rarely reported (in accordance with the results of Efficace et al. (2015) systematic review [64] about RCTs across cancer patients), making it hard to determine whether open-label trials could have influenced the responses to the questionnaires.

Finally, mains weaknesses associated with HRQOL in RCTs about patients treated with ICIs are the same as those described in cancer RCTs (the statement of a PRO hypothesis, the documentation of methods for PRO data collection, etc.). However, in immunotherapy RCTs, the “adequacy of domains covered” criterion is no satisfy because none of the questionnaire used in these RCTs covered, at least, the main HRQOL dimensions relevant according to the specific research question, namely irAEs. There is a need to develop standard methods for evaluating HRQOL for patients treated with ICIs. But HRQOL and irAEs are different according to cancer type and/or ICIs administered. We propose that HRQOL about patients treated with ICIs is assessed with the common cancer HRQOL assessment tools (like QLQ-C30, FACT-G), a specific module according to the type of cancer (as QLQ-LC13, QLQ-H&N35, LCSS) and a specific module for evaluate irAEs according to ICIs administered. At present, in the absence of HRQOL tool for patients treated with ICIs and for studying HRQOL more rigorously:

1. we propose that HRQOL, in RCTs about patients treated with ICIs, was a primary endpoint not secondary or exploratory. Indeed, HRQOL endpoints are increasingly being used in cancer RCTs but often in secondary endpoint [64,81]. However, regardless of cancer type, quality of reporting was typically higher in RCTs where HRQOL were primary endpoints [81];
2. As already mentioned, in our research, none of RCTs included an *a priori* hypotheses for the expected HRQOL outcomes whereas a clear hypothesis is an essential requirement for an appropriate study design, the choice of the instrument, etc. [20, 9, 22, 25]. So, in future RCTs about patients treated with ICIs a hypothesis must be included;
3. In our research, HRQOL data was judged as high quality in ten RCTS. Because methodological rigor is essential to the conduct and reporting of RCTs [64], some recommendations have been established with the aim to facilitate adherence with key methodological aspects and to increase the transparency and consistency of PRO reporting in RCTs (for example, the Consolidated Standards of Reporting Trials (CONSORT) group published the CONSORT PRO extension in 2013; the Minimum Standard Checklist for evaluating HRQOL Outcomes in Cancer Clinical Trials by Efficace et al. [25]). In cancer RCTs, the overall level of reporting according to the new CONSORT PRO extension was poor. However, adherence to the CONSORT PRO extension was higher in RCTs in which PROs were included as primary endpoints versus RCTs with PROs as secondary outcomes [65]. All cancer RCTs, including RCTs with patients treated with ICIs, must implement of the CONSRT PRO extension for example;
4. Patient-generated outcome measures have been developed in an effort to capture the individualistic nature of HRQOL. These measures differ from traditional HRQOL instruments in that they allow patients to individually define HRQOL domains or weights [82]. Different tools are available: Schedule for the Evaluation of Individual Quality of Life (SEIQoL), Repertory Grid, and Asthma Quality of Life Questionnaire (AQLQ) or Patient-Generated Index (PGI). For Patel et al., (2003), patient-generated outcome measures appear to be useful primarily in complementing traditional HRQOL measures, guiding individual patient treatment decisions, and assisting the design of new measures [82]. For example, the PGI is designed to both ask and document HRQOL concerns [83]. The PGI consists of three stages in which patients: i) self-identify the most important areas or activities of their lives affected by their condition; ii) score the degree to which each area is affected; and iii) allocate points among the items listed to represent the amount in which they would like each area improved [84]. A recent study provides evidence that the PGI would be a good measure for cancer patients and clinicians to use together [83]. Tang et al. (2014) systematic review aims to identify current literature in which PGI has been used as a tool to assess quality of life in cancer patients [84]. An overarching theme observed in these studies highlighted the concerns mentioned by patients that were not targeted or detected by standardized quality of life measures [84]. In HRQOL RCTs about patients treated with ICIs, patient-generated outcomes measures could be used to identify areas of cancer which are not evaluated by common cancer tools and that require attention and monitor changing needs

### Study limitations

Our systematic review has several limitations. We focused only on RCTs published in French and English, but as the most important RCTs tend to be published in English journals, we believe that we have included the most significant trials. Another limitation is that we based our work on the information that the clinical community could access and only with the literature that reported publishing HRQOL. Unpublished RCTs and RCTs including HRQOL endpoint by design, but that had not published their results, protocols, or statistical analysis plans, were excluded. Our review was therefore subject to publication bias. Furthermore, though we used subjective judgments for several assessment criteria, each criterion was independently assessed by two reviewers under the supervision of a third researcher. We therefore believe that our results are reproducible and consistent. Lastly, since checkpoint inhibition is associated with a unique spectrum of side effects that are not assessed with the HRQOL measure used in the RCTs identified, the HRQOL domains covered by these questionnaires were not adequate for all the trials. As such, it is difficult to explore the value added by these measures to clinical decision-making in a trial setting.

## Conclusion

To the best of our knowledge, we conducted one of the first systematic reviews aimed at investigating the HRQOL assessment methods used in RCTs for cancer patients treated with ICIs. HRQOL quantifies how a patient feels or functions, provides an economic basis for decision-making and contributes to clinical decision-making. As such, HRQOL is playing a more prominent role as an important endpoint in cancer clinical trials. The quality of the measurements and interpretation of results is crucial. Previous findings suggest that ICIs maintain HRQOL compared with standard treatments. However, these results must be interpreted with caution in view of a number of potential weaknesses associated with the RCTs included (such as open-label trials, under-reporting of HRQOL research hypotheses, instruments not validated for patients treated with immunotherapy) and insufficient guidance, as well as the lack of internationally recognised standard methods for analysing and reporting HRQOL. Hence, there is a strong need to develop standard methods for evaluating HRQOL for patients treated with ICIs. A better methodology would lead to a more efficient understanding of HRQOL outcomes.

## Supporting information

S1 Prisma checklist (DOC)

S1 Search algorithm (DOCX)

S1 Table Minimum Standard Checklist for evaluating HRQOL Outcomes in Cancer Clinical Trials. *If a study explicitly states an exploratory HRQOL evaluation. ¶ If the HRQOL measure is validated in the same population as the one of the trial.
(DOCX)

S2 Table Assessment methods for HRQOL. HRQOL = heath-related quality of life. EORTC QLQ-C30 = European Organisation for Research and Treatment of Cancer Core (EORTC) Quality of Life Questionnaire. EQ-5D = European Quality of Life 5 Dimension. EORTC QLQ-LC13 = EORTC Quality of Life 13-item Lung Cancer-specific Questionnaire. EORTC QLQ-H&N35 = EORTC module for Head & Neck cancer. LCSS = Lung Cancer Symptom Scale. FACT-G = Functional Assessment of Cancer Therapy-General. N/A = Not Available.
(DOCX)
