# Supplementary material for: Health-related quality of life in cancer patients treated with immune checkpoint inhibitors: A systematic review on reporting of methods in randomized controlled trials
Source: PLoS One. 2020 Jan 24;15(1):e0227344. doi: 10.1371/journal.pone.0227344 (PMC6980610; doi:10.1371/journal.pone.0227344)
Supplement: S1 Search algorithm — (DOCX) [file pone.0227344.s002.docx]

**S1 Search algorithm.**

("quality of life"[MeSH Terms] OR quality of life[Text Word]) AND (("atezolizumab"[Supplementary Concept] OR "atezolizumab"[All Fields]) OR ("nivolumab"[Supplementary Concept] OR "nivolumab"[All Fields]) OR ("pembrolizumab"[Supplementary Concept] OR "pembrolizumab"[All Fields]) OR ("ipilimumab"[MeSH Terms] OR "ipilimumab"[All Fields]) OR ("avelumab"[Supplementary Concept] OR "avelumab"[All Fields]) OR ("durvalumab"[Supplementary Concept] OR "durvalumab"[All Fields])) AND ("neoplasms"[MeSH Terms] OR "neoplasms"[All Fields] OR "cancer"[All Fields]).
