# Supplementary material for: Health-related quality of life in cancer patients treated with immune checkpoint inhibitors: A systematic review on reporting of methods in randomized controlled trials
Source: PLoS One. 2020 Jan 24;15(1):e0227344. doi: 10.1371/journal.pone.0227344 (PMC6980610; doi:10.1371/journal.pone.0227344)
Supplement: S1 Table — *If a study explicitly states an exploratory HRQOL evaluation. ¶ If the HRQOL measure is validated in the same population as the one of the trial. (DOCX) [file pone.0227344.s003.docx]

| HRQOL Issue | Answer | | | Description |
| --- | --- | --- | --- | --- |
| **Conceptual** |  |  |  |  |
| A priori hypothesis stated | Yes | No | N/A* | Assessed whether authors had a predefined HRQOL end point and/or stated expected changes because of the specific treatment. |
| Rationale for instrument reported | Yes | No |  | Assessed whether authors gave a rationale for using a specific HRQOL measure. |
| **Measurement** |  |  |  |  |
| Psychometric properties reported | Yes | No |  | Assessed whether a previously validated measure was used or psychometric properties were reported or referenced in the article. |
| Cultural validity verified* | Yes | No | N/A¶ | Assessed whether the measure was validated for the specific study population. |
| Adequacy of domains covered** | Yes | No |  | Assessed whether the measure covered, at least, the main HRQOL dimensions relevant for a generic cancer population and/or according to the specific research question. |
| **Methodology** |  |  |  |  |
| Instrument administration reported | Yes | No |  | Assessed whether authors specified who and/or in which clinical setting the HRQOL instrument was administered. |
| Baseline compliance reported | Yes | No |  | Assessed whether authors reported the number of patients providing an HRQOL assessment before the start of treatment. |
| Timing of assessments documented | Yes | No |  | Assessed whether authors specified the HRQOL timing of assessment during the trial. |
| Missing data documented | Yes | No |  | Assessed whether authors gave some details on HRQOL missing data during the trial. |
| **Interpretation** |  |  |  |  |
| Clinical significance addressed | Yes | No |  | This refers to the discussion of HRQOL data being clinically significant from a patient’s perspective and not simply statistically significant. |
| Presentation of results in general | Yes | No |  | Assessed whether authors discussed the HRQOL outcomes, giving any comments regardless of the results (either expected or not). |

**S2 Table. Minimum Standard Checklist for evaluating HRQOL Outcomes in Cancer Clinical Trials** [25].

*To satisfy the “cultural validity verified” criterion, the study needed to use an HRQOL instrument validated for cancer patients.

**To satisfy the “adequacy of domains covered” criterion in the case of a trial with a predefined HRQOL endpoint, we assessed as ‘yes’ if the domain related to the specific research question of the trial covered by the measure used. The trial research question may be unanswered if an HRQOL measure is not sensitive enough to detect HRQOL changes for the specific target population of the study [25].
