# Supplementary material for: Health-related quality of life in cancer patients treated with immune checkpoint inhibitors: A systematic review on reporting of methods in randomized controlled trials
Source: PLoS One. 2020 Jan 24;15(1):e0227344. doi: 10.1371/journal.pone.0227344 (PMC6980610; doi:10.1371/journal.pone.0227344)
Supplement: S2 Table — HRQOL = heath-related quality of life. EORTC QLQ-C30 = European Organisation for Research and Treatment of Cancer Core (EORTC) Quality of Life Questionnaire. EQ-5D = European Quality of Life 5 Dimension. EORTC QLQ-LC13 = EORTC Quality of Life 13-item Lung Cancer-specific Questionnaire. EORTC QLQ-H&N35 = EORTC module for Head & Neck cancer. LCSS = Lung Cancer Symptom Scale. FACT-G = Functional Assessment of Cancer Therapy-General. N/A = Not Available. (DOCX) [file pone.0227344.s004.docx]

| **S3 Table. Assessment methods for HRQOL.** | | | | | | | | | | | | | | | | |
| --- | --- | --- | --- | --- | --- | --- | --- | --- | --- | --- | --- | --- | --- | --- | --- | --- |
| **First author year**  **Main publication year** | **HRQOL endpoint** | **HRQOL assessment method** | **Conceptual** | |  | **Measurement** | | |  | **Methodology** | | | |  | **Interpretation** | |
|  |  |  | A priori hypothesis stated | Rational for instruments reported |  | Psychometric properties reported | Cultural validity verified | Adequacy of domains covered |  | Instrument administration reported | Baseline compliance reported | Timing of assessment reported | Missing data documented |  | Clinical significance addressed | Presentation of results in general |
| Bordoni 2018  Rittmeyer 2017 | Secondary | EORTC QLQ-C30  EORTC QLQ-LC13 | Yes | Yes |  | Yes | Yes  Yes | No |  | No | Yes | Yes | Yes |  | Yes | Yes |
| Brahmer 2017  Reck 2016 | Exploratory | EORTC QLQ-C30  EORTC QLQ-LC13  EQ-5D-3L | N/A | Yes |  | Yes | Yes  Yes  No | No |  | Yes | Yes | Yes | Yes |  | Yes | Yes |
| Cella 2016  Motzer 2015 | Secondary | FKSI-DRS  EQ-5D-3L | Yes | Yes |  | Yes | Yes  No | No |  | No | Yes | Yes | Yes |  | Yes | Yes |
| Cella 2019  Motzer 2018 | Exploratory | FKSI-19  FACT-G  EQ-5D-3L | N/A | Yes |  | Yes | Yes  Yes  No | No |  | Yes | Yes | Yes | Yes |  | Yes | Yes |
| Coens 2017  Eggermont 2015 | Secondary | EORTC QLQ-C30 | Yes | Yes |  | Yes | Yes | No |  | No | Yes | Yes | Yes |  | Yes | Yes |
| Harrington 2017  Ferris 2016 | Exploratory | EORTC QLQ-C30  EORTC QLQ-H&N35  EQ-5D-3L | N/A | Yes |  | Yes | Yes  Yes  No | No |  | Yes | Yes | Yes | Yes |  | Yes | Yes |
| Larkin 2018  Weber 2015 | Secondary | EORTC QLQ-C30  EQ-5D | No | No |  | Yes | Yes  No | No |  | No | No | Yes | Yes |  | No | No |
| Long 2016  Robert 2015 | Secondary | EORTC QLQ-C30  EQ-5D | No | Yes |  | Yes | Yes  No | No |  | Yes | Yes | Yes | Yes |  | Yes | Yes |
| Petrella 2017  Robert 2015 | Secondary | EORTC QLQ-C30  EQ-5D | Yes | Yes |  | Yes | Yes  No | No |  | Yes | Yes | Yes | Yes |  | Yes | Yes |
| Reck 2018  Brahmer 2015 | Secondary | LCSS  EQ-5D | Yes | Yes |  | Yes | Yes  No | No |  | Yes | Yes | Yes | Yes |  | Yes | Yes |
| Revicki 2012  Hodi 2010 | Unclear | EORTC QLQ-C30 | Unclear | Yes |  | Yes | Yes | No |  | Yes | Yes | Yes | Yes |  | Yes | Yes |
| Schadendorf 2016  Ribas 2015 | Exploratory | EORTC QLQ-C30 | N/A | Yes |  | No | Yes | No |  | No | Yes | Yes | Yes |  | Yes | Yes |
| Schadendorf 2017  Larkin 2015 | Secondary | EORTC QLQ-C30  EQ-5D | No | Yes |  | Yes | Yes  No | No |  | No | Yes | Yes | Yes |  | Yes | Yes |
| Vaughn 2018  Bellmunt 2017 | Exploratory | EORTC QLQ-C30  EQ-5D | N/A | Yes |  | No | Yes  No | No |  | Yes | Yes | Yes | Yes |  | Yes | Yes |
| Weber 2017  Same | Secondary | EORTC QLQ-C30  EQ-5D | No | No |  | Yes | Yes  No | No |  | No | Yes | Yes | Yes |  | No | No |
|  | | | | | | | | | | | | | | | | |
